# The effect of different implant programs on beef × dairy steer feedlot growth performance and carcass characteristics

**DOI:** 10.1093/tas/txad124

**Published:** 2023-11-11

**Authors:** K R Wesley, A B Word, C L Maxwell, B P Holland, K J Karr, J P Hutcheson, L A J Walter, B J Johnson

**Affiliations:** Department of Animal and Food Sciences, Texas Tech University, Lubbock, TX 79409; Cactus Research, Amarillo, TX 79101; Cactus Research, Amarillo, TX 79101; Cactus Research, Amarillo, TX 79101; Cactus Research, Amarillo, TX 79101; Merck Animal Health, Madison, NJ 07940; Merck Animal Health, Madison, NJ 07940; Department of Animal and Food Sciences, Texas Tech University, Lubbock, TX 79409

**Keywords:** beef × dairy, estradiol-17β, implant, performance, steers, trenbolone acetate

## Abstract

The objective of this study was to evaluate potency and timing of trenbolone acetate (**TBA**) administration on live performance and carcass characteristics of beef × dairy steers. A total of 6,895 beef × dairy steers [initial body weight (**BW**) = 157 ± 5.2 kg] were allotted into 30 pens, with pen as the experimental unit. Each pen was randomly assigned one of three implant treatments: 1) Revalor-IS (**IS**) at d 0, IS at d 80, and Revalor-XS (**XS**) at d 160 (**IS/IS/XS**); 2) Ralgro at d 0, IS at d 80, and XS at d 160 (**Ral/IS/XS**); or 3) Encore at d 0 and XS at d 160 (**Enc/XS**). Steers were blocked by arrival date, each pen was terminally sorted in three ways at 257 ± 22 days on feed and harvested at 329 ± 25 days on feed. For live and carcass outcomes, fixed effect of implant treatment and random effect of block was evaluated. Data are reported on a deads and removals out basis. Removals, morbidity, and mortality were similar (*P* ≥ 0.45). Steers administered TBA prior to d 160 were 5.8 kg heavier (*P* = 0.03) than Enc/XS steers at d 160. Final BW was not different (*P* = 0.78). Early administration of a TBA-containing implant resulted in an increased prevalence of bullers [2.40%, 5.18%, 6.86% (for Enc/XS, Ral/IS/XS, and IS/IS/XS) respectively; *P* < 0.01]. Dry matter intake (**DMI**) was 2.3% greater (*P* < 0.01) in steers administered Enc/XS compared to IS/IS/XS; however, DMI as a percentage of BW, average daily gain, and feed efficiency were not different (*P* ≥ 0.12). Dressing percentage, hot carcass weight, heavy carcass occurrence, Longissimus muscle area, and 12th rib fat thickness were similar among all steers (*P* ≥ 0.28). Marbling score tended to be greatest for Enc/XS and Ral/IS/XS (*P* = 0.09). Enc/XS graded a greater proportion of USDA Prime and fewer USDA Select carcasses than IS/IS/XS (*P* < 0.05). Enc/XS and Ral/IS/XS tended (*P* = 0.09) to have more USDA Yield Grade (**YG**) 1 carcasses. While delayed administration or decreased total potency of TBA-containing implants may decrease buller incidence and improve Quality Grade, few differences were observed in live or carcass outcomes.

## Teaser

Increased potency and earlier administration of TBA-containing implants increased prevalence of bullers, but implant treatment did not affect live growth performance outcomes.

Quality Grade improved and empty body fat percentage was greater in steers administered the lowest potency and latest administration of TBA implants, with no other differences for carcass performance.

## INTRODUCTION

Historically, the dairy industry produced 50% replacement heifers and 50% bull calves. Bull calves were viewed as byproducts of the dairy industry and raised for beef in conventional cattle feeding operations. In the contemporary feedlot, a year-round supply of Holstein steers can mitigate seasonal fluctuations in beef calf supply, improve traceability throughout the beef production system, and enhance marbling and eating experience (Duff and McMurphy, 2007). However, Holstein steers have larger frame sizes, lighter placement weights, and longer days on feed associated with a greater prevalence of liver abscesses and poor carcass cutability compared with beef steers (Duff and McMurphy, 2007; Kayser et al., 2019). These negative carcass characteristics have recently resulted in beef processors discounting or banning Holstein steers from harvest. Through the use of beef semen in Holstein females, the industry has increased beef × dairy cattle supplied to beef processors, while acknowledging benefits of feeding dairy-sourced steers in the feedlot (De Vries et al., 2008) and increased the prevalence of beef × dairy calves in feedlots. While research is currently being conducted to aid producers in determining appropriate management of beef × dairy cattle, very little data are currently available in the published literature, and more research is necessary.

Growth-promoting implants are administered to more than 92% of cattle in a feedlot setting (NAHMS, 2013) for improved production and efficiency (Perry et al., 1991; Duckett and Andrae, 2001). The combination trenbolone acetate (**TBA**) and estradiol implants increased the expression of genes associated with muscle tissue growth (Parr et al., 2014; Smith et al., 2017, 2019). Based on previous literature, there appears to be an effect of TBA administration potency and timing on the live and carcass performance of fed, pure-bred Holstein cattle which is not fully understood (Scheffler et al., 2003; Torrentera et al., 2017; Kayser et al., 2019; Quinn et al., 2020) and could be extrapolated to beef × dairy steers. Therefore, the objective of this study was to evaluate the effects of timing and total potency of TBA administration on bulling outcomes, live performance, and carcass characteristics of beef × dairy steers.

## MATERIALS AND METHODS

### Study Animals and Location

Live animal care and management followed guidelines provided in *Guide for the Care and Use of Agricultural Animals in Research and Teaching, 4th Edition* (FASS, 2020). All steers were harvested at a commercial abattoir.

This study was conducted at a commercial feedyard in the Texas Panhandle from August 2020 to January 2022. Predominantly black-hided, beef-type sire × dairy-type dam steers (*n* = 6,981) were shipped approximately 1,910 km from a commercial calf ranch in California. Upon arrival, steers were acclimated to the environment with access to hay and water for at least 24 h prior to processing. A total of 6,895 steers [body weight (**BW**) = 157 ± 5.2 kg] were enrolled in the study. A total 86 animals were rejected at study enrollment at arrival because they were bulls, heifers, greater than 50% blind, or were predominantly white or red-hided and assumed to be of different sire breed than desired.

### Treatment Allocation and Study Design

Steers were excluded from the trial at enrollment if deemed unlike the desired breed type, visually ill, or lame. Steers were blocked by arrival time, where each block (*n* = 10) contained one replication of each implant treatment. Each pen contained 210 to 250 steers on d 0, and pens within a block were filled to the same *n* ± 1 steer. Within each group of three consecutive steers in the chute at processing, steers were randomly assigned to one of three study pens in the block using a computer-generated random number assignment. Within blocks, pens were randomly assigned to one of the three treatments detailed below, at d 0.

1. IS/IS/XS: Steers were administered Revalor-IS (**IS**) on both d 0 and d 80, and Revalor-XS (**XS**) on d 160.
2. Ral/IS/XS: Steers were administered Ralgro on d 0, IS on d 80, and XS on d 160.
3. Enc/XS: Steers were administered Encore on d 0 and XS on d 160.

Each Ralgro (Merck Animal Health, Madison, NJ) implant provides 36 mg of zeranol within three condensed pellets with an estimated release over 70 to 120 days (Crawford, 2021). The Revalor-IS (Merck Animal Health) implant provides a combination of 80 mg of TBA and 16 mg of estradiol within four condensed pellets and had been noted effective for up to 130 days (Crawford, 2021). An Encore (Elanco Animal Health, Greenfield, IN) implant is coated in a silastic rubber and provides 43.9 mg of estradiol released over a period of 400 days. Finally, a Revalor-XS (Merck Animal Health) implant is made up of four uncoated pellets that begin to release 80 mg of TBA and 16 mg of estradiol immediately after administration, while the remaining six coated pellets will begin to degrade at approximately 70 days post-implantation to provide 120 mg of TBA and 24 mg of estradiol for the remaining 130 days for a total of 200 mg of TBA and 40 mg of estradiol released over 200 days (Parr et al., 2014).

Steers in each pen had projected harvest date assigned at arrival by an internal, proprietary projection program in addition to visual evaluation of body condition and BW by the general manager at terminal sort. Prior to the projected harvest date, each pen was sorted (257 ± 22 days on feed) into light, medium, or heavy weight pens using the proprietary sorting technology from Performance Cattle Company (Amarillo, TX). All three pens within a block were terminally sorted on the same day, resulting in three replications of each implant treatment per block. After terminal sort, pens contained between 57 to 84 steers each. All pens in the same terminal sort group within a block were shipped approximately 177 km on the same day to a commercial abattoir, equating to three shipping days (one day for light, one day for medium, and one day for heavy weight sort) for each block.

### Animal Management

Steers were housed in soil-surfaced, outdoor pens allowing a minimum of 12.5 m^2^ pen space to each steer during the beginning of the trial and following terminal sort. The concrete feed bunk spanned the width of the pen, approximately 18 cm of bunk space per steer was provided, feed was delivered three times daily, and each pen was equipped with a center-pen constant-flow concrete water tank.

On d 0, steers were vaccinated against clostridial diseases (Vision 7, Merck Animal Health), viral respiratory pathogens (Vista 5 LS, Nasalgen IP, Once PMH SQ, Merck Animal Health), treated for internal and external parasites (Dectomax, Zoetis Animal Health, Parsippany, NJ), and received antimicrobial metaphylaxis (Zuprevo, Merck Animal Health). On d 160, all steers were administered respiratory (Titanium IBR LP; Elanco Animal Health) and clostridial vaccines (Vision CD; Merck Animal Health).

Steers were observed daily for general health and well-being, and unusual observations were recorded. Animals exhibiting signs of illness or injury were taken to a hospital facility for further evaluation and treatment, if necessary. Animals visually evaluated as not growing at the same rate as pen-mates, non-responsive to two bovine respiratory disease treatments not including metaphylaxis, injured animals, or those not receiving an implant within ±3 days of pen mates were removed from the experiment. Steers that died or were euthanized during the experiment had field necropsy performed, and cause of death was recorded.

Steers were identified and removed from the home pen as bullers if: bulled excessively, appeared fatigued or depressed through visual observation, and exhibited signs of bulling activity such as removal of hair or abrasions on the tail head and hips. Those identified and removed as bullers were placed in a buller pen until the next pen event (i.e., reimplant, terminal sort, shipping) when all bullers in that lot were reintroduced to the home pen. If a steer was returned to the home pen after an initial buller removal and relapsed, he was again removed and placed in the buller pen until the next pen event.

Samples of each diet (Table 1) were collected daily from the feed bunk and subsamples dried at 100 °C for 24 h to calculate daily dry matter intake. An additional sample from each diet was collected monthly and submitted to Servi-Tech Laboratories (Amarillo, TX) for analysis. The starter diet for all treatments was RAMP (Cargill Corn Milling, Bovina, TX). Transition to the finishing diet was done using a two-ration approach where systematic replacement of the daily feed call of RAMP was replaced with a finisher diet. Finisher diets were prepared in the on-site feed mill, micro-ingredients were weighed using a Micro Machine (Micro Technologies, Amarillo, TX), and added to the feed batch. Monensin (Rumensin, Elanco Animal Health) was included in the RAMP [20 g/ton; 100% dry matter (**DM**) basis] and finisher diets (42.1 g/ton), and tylosin phosphate (Tylan, Elanco Animal Health) was included in the diet at 8.9 g/ton throughout the experiment. All steers, regardless of sort group, were fed ractopamine HCl (Optaflexx, Elanco Animal Health) for 29 days prior to slaughter at 400 mg/steer/d.

**Table 1.** Diet formulation and analyzed composition calculated by weighted average of days fed

Item	Inclusion, %
Formulation, % of DM	
Steam-flaked corn	53.2
Wet corn gluten feed	18.7
Wet distillers grains	14.7
Roughage^1^	8.1
Liquid blended fat^2^	4.0
Corn condensed distiller’s solubles	1.4
Micro ingredients^3^	0.03
Analyzed composition^4^	
Diet DM, %	61.1 ± 3.0
CP, %	15.1 ± 0.8
NDF^5^, %	21.7 ± 2.2
Crude fat, %	5.39 ± 0.65
Calcium, %	0.85 ± 0.14
Phosp2, horus, %	0.56 ± 0.04
Magnesium, %	0.25 ± 0.02
Potassium, %	0.92 ± 0.09

^1^Roughage ingredients consisted of corn silage from August 10, 2020 to October 2, 2020, ground cotton burrs from October 3, 2020 to Feburary 9, 2021, corn stalks from Feburary 10, 2021 to November 31, 2021, and whole cotton burrs December 1, 2021 to January 21, 2022.

^2^Liquid-blended fat was made from soybean sources with carriers, approximately 42% fat.

^3^Micro-ingredients included vitamin A at 544 IU/kg, vitamin D at 54 IU/kg, Rumensin 90 at 42.1 g/ton, Tylan 100 at 7.5 g/ton, and Optaflexx during the final 29 days prior to harvest.

^4^All values except diet DM are presented on a DM basis. Analyzed by Servi-Tech Laboratories, Amarillo, TX. *n* = 27 samples analyzed. Values listed as percentage ± standard deviation.

^5^Formulated to provide a minimum 19% NDF in the diet.

### Data Collection

Steers were group-weighed by pen using a platform scale on study d 0, d 160, and the morning of shipment for calculation of live growth performance. Body weight (BW) was measured prior to morning feeding and a 4% shrink was applied to d 160 and final BW. Shrink was not applied to d 0 BW due to prolonged duration of transportation.

Trained personnel from the Beef Carcass Research Center (**BCRC**; West Texas A&M University; Canyon, TX) recorded individual animal ear tag numbers in the sequence of harvest and affixed a harvest sequence number to each carcass. Plant carcass ID and hot carcass weight (**HCW**) were recorded and verified by carcass sequence number. Livers were scored for presence and severity of abscesses (none, A, A-, A+) using the Elanco Liver Scoring System and for presence of other abnormalities (adhesions, cirrhosis, telangiectasis, flukes). Carcasses were graded after an approximately 36 h chill. Processing plant records were utilized to match carcass ID recorded by BCRC to assigned United States Department of Agriculture (**USDA**) Quality Grade (assigned by USDA Grader), yield grade (assigned by camera system), HCW, and camera measurements. Data were collected for 86 pens in this manner.

For four pens, data were not recorded by the BCRC due to a communication error. For these pens, processing plant records were utilized to assign carcasses to lots, and liver scores were not available. Two terminal sort pens were from IS/IS/XS and two terminal sort pens were from Enc/XS; three pens were from block 4 and one pen was from block 8.

### Statistical Analysis

Dressing percentage for each pen was calculated as the mean HCW/ mean shrunk final BW × 100. Carcass adjusted final BW was calculated as mean final BW per pen divided by 63.30%, the average dressing percentage across all treatments. Data were analyzed as a randomized complete block design. The model included fixed effects of implant treatment and random effects of block. Data were analyzed on an alloment pen level for live performance, experimental units (**EU**) = 30, and on a terminal sort pen level for carcass variables EU = 90. Morbidity, mortality, removals, and buller data were analyzed on an individual steer basis using logistic regression with fixed effect of implant treatment and random effect of block. Aggregated pen-level data were analyzed using the lm function in the stats package and Anova in the car package (Fox and Weisberg, 2019) of RStudio 4.1.1 (RStudio; Boston, MA). Presented means were calculated by using pairwise comparisons in the emmeans package (Lenth, 2021). When outcomes were significant for main effects of implant treatment, means were separated using cld (multcomp package; Hothorn et al., 2008) with a Tukey adjustment and 95% confidence level. Heavy carcasses, liver outcomes, USDA Quality Grade, and USDA Yield Grade outcomes were analyzed using logistic regression (glm in the stats package; R Core Team, 2021) with fixed and random effects as described above.

## RESULTS AND DISCUSSION

### Removals and Mortalities

Mortality, morbidity, and removal outcomes for this study are presented in Table 2. No differences (*P* ≥ 0.13) of implant treatment were detected for overall or subcategory of morbidity. In the present study, total morbidity averaged 13.5% across treatments with respiratory treatments responsible for more than 84% of total morbidity (data not shown). The level of total morbidity is congruent with Kayser et al. (2019) who reported up to 14% morbidity in a similar large-pen implant study with dairy influenced steers that were on feed for 360 days.

**Table 2.** Morbidity and mortality of beef × dairy steers administered differing implant programs.

Item	Treatment^1^				*P*-value
	IS/IS/XS	Ral/IS/XS	Enc/XS	SEM	Implant
Steers, *n*	2299	2299	2297		
Morbidity					
Overall, %	13.41	13.76	13.45	0.989	0.95
Respiratory, %	11.54	11.67	11.32	0.922	0.95
Digestive, %	0.74	0.92	0.77	0.188	0.70
Other^2^, %	1.13	1.17	1.36	0.276	0.77
Removals					
Overall, %	3.32	2.88	2.69	0.367	0.45
Respiratory, %	0.43	0.43	0.45	0.177	0.99
Digestive, %	0.14	0.09	0.00	0.053	0.13
Other health^3^, %	2.10	1.99	2.00	0.371	0.97
Other non-health^4^, %	0.61	0.43	0.23	0.120	0.02
Mortality					
Overall, %	9.32	8.69	9.50	0.700	0.53
Respiratory, %	5.09	4.23	4.78	0.598	0.28
Digestive, %	3.67	4.16	4.29	0.499	0.44
Other^5^, %	0.56	0.30	0.43	0.156	0.30
Buller Pulls					
All pulls, %	6.86^a^	5.18^ab^	2.40^b^	0.872	<0.01
Pulls D0–D80, %	0.18	0.00	0.09	0.079	0.25
Pulls D80–D160, %	2.70^a^	0.48^b^	0.21^b^	0.270	<0.01
Pulls D160–End, %	3.97^a^	4.71^a^	2.11^b^	0.743	<0.01
First pulls, %	5.27^a^	4.16^a^	1.90^b^	0.699	<0.01
Pulls D0–D80, %	0.18	0.00	0.09	0.079	0.25
Pulls D80–D160, %	2.30^a^	0.31^b^	0.04^b^	0.258	<0.01
Pulls D160–End, %	2.78^ab^	3.85^a^	1.77^b^	0.589	0.03

^1^IS/IS/XS = Revalor-IS (80 mg TBA and 16 mg estradiol; Merck Animal Health) on d 0 and d 80, and Revalor-XS (200 mg TBA and 40 mg estradiol; Merck Animal Health) on d 160; Ral/IS/XS = Ralgro (36 mg zeranol; Merck Animal Health) on d 0, Revalor-IS on d 80, and Revalor-XS on d 160; Enc/XS = Encore (43.9 mg estradiol; Elanco Animal Health) on d 0 and Revalor-XS on d 160.

^2^Other reasons for morbidity include cripple, footrot, waterbelly, and diagnosis not specified.

^3^Other reasons for removal related to health-included abscess, hardware, lameness, and non-doers.

^4^Other reasons for removal not related to health-included animals not receiving an implant within ± 3 days of penmates.

^5^Mortality due to reasons other than respiratory or digestive outcomes include primarily euthanasia and hardware disease.

^a,b,c^Means in a row with varying superscripts differ (*P *< 0.05).

No differences (*P* ≥ 0.13) of implant treatment for animal removal overall or in any subcategory analyzed were observed. Non-health related removals indicated steers that were not implanted at the same time as pen-mates.

No differences (*P* ≥ 0.28) were observed for mortality related to implant treatment. Mortality rates in the present study were greater than the expected 4% to 7% observed by Kayser et al. (2019) and Quinn et al. (2020) for Holstein steers fed for similar days on feed. In the present study, 70% of deaths occurred prior to 120 days on feed and 48% of removals occurred between 200 and 300 days on feed. However, Buchanan et al. (2020) reports mortality rates of up to 9.5% for calf-fed Holstein steers, indicating the 9% death loss in this study is not unprecedented. Respiratory death loss was responsible for 51% of the death loss in the current study, while digestive death loss was the second mortality cause. Likewise, Edwards (1996) and Vogel et al. (2015) reported respiratory disease accounted for more than 50% and 46% of the mortality in feedlot cattle, respectively. Respiratory treatments accounted for 84% of total morbidity, which suggests the morbidity percentage for treated respiratory illness is approximately two times greater than respiratory mortality losses. Moreover, Engler et al. (2014) reported a 1% increase in mortality was associated with a $20.07 per head loss on marketed animals, suggesting sizable economic impacts in the present study.

Although researchers acknowledge an increased rate of mortality in the present study compared to those observed throughout the industry, several factors were considered. Babcock et al. (2013) reported the greatest risk of mortality and culling in cattle entering the feedlot at less than 182 kg; these cattle reached a peak mortality and culling rate of 9.9% with an 8.4%–11.6% confidence interval. Duff and McMurphy (2007) indicated Holstein steers are likely at risk for more metabolic disorders and liver abscesses than native breed types due to extended days on feed and increased consumption of high-concentrate diets. Garrett (1971) reported a 25% greater intake for Holstein steers compared to Hereford steers due to greater energy requirements. Moreover, death loss due to digestive outcomes has been observed at a high percentage of mortality than those observed in beef cattle (Grant et al., 1993), and Zhang et al. (2019) reported the greatest portion of mortality in Holstein cattle was attributed to digestive diseases. Buda et al. (2020) also reported a 0.634 higher death loss coefficient for Holstein cattle than typical steers. These studies reveal that light-weight Holstein-influenced cattle are at a high risk for negative morbidity and mortality outcomes, similar to those observed in this study. Nonetheless, these studies also indicate that feedyard mortality may vary greatly, despite the best management practices.

**Figure 1.**
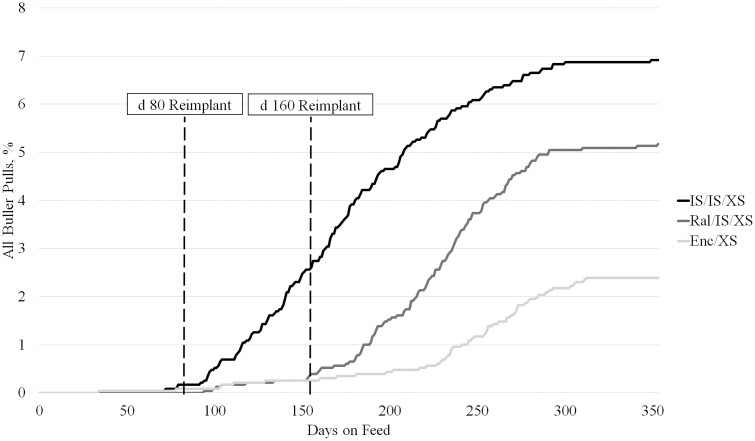
All buller pulls over days on feed of beef × dairy steer administered different implant programs and terminally sorted. First, second, third, and fourth pulls are represented in the above chart. Implant treatments consisted of Revalor-IS (80 mg TBA + 16 mg E_2_; IS; Merck Animal Health, Madison, NJ) administered on d 0 and d 80, and Revalor-XS (200 mg TBA + 20 mg E_2_; XS; Merck Animal Health) on d 160 (**IS/IS/XS**); Ralgro (36 mg zeranol; Merck Animal Health) administered at d 0, IS at d 80, and XS at d 160 (**Ral/IS/XS**); or Encore (43.9 mg E_2_; Elanco Animal Health, Greenfield, IN) administered at d 0 and XS at d 160 (**Enc/XS**). Steers were terminally sorted within block and treatment into light, medium, or heavy groups between d 244 and d 300.

Steers identified as bullers are often associated with economic losses due to removal from the home pen, increased management costs, decreased performance compared to pen mates, and heightened potential for injury (Ulbrich, 1981). Furthermore, Ulbrich (1981) speculates the administration of a growth promoting implant may increase the occurrence or severity of bulling occurrences. All buller pulls, regardless of first, second, third, or fourth pull, were affected by implant treatment (*P* < 0.01); IS/IS/XS steers were 1.9 times more likely to be pulled compared to Enc/XS steers, while Ral/IS/XS steers were intermediate (6.86% vs. 2.40% and 5.18%; Figure 1). Similarly, first pulls for bulling activity were 2.8 and 2.2 times greater (*P* < 0.01) for steers administered IS/IS/XS and Ral/IS/XS than those given Enc/XS, respectively (5.27% and 4.16% vs. 1.90%).

**Figure 2.**
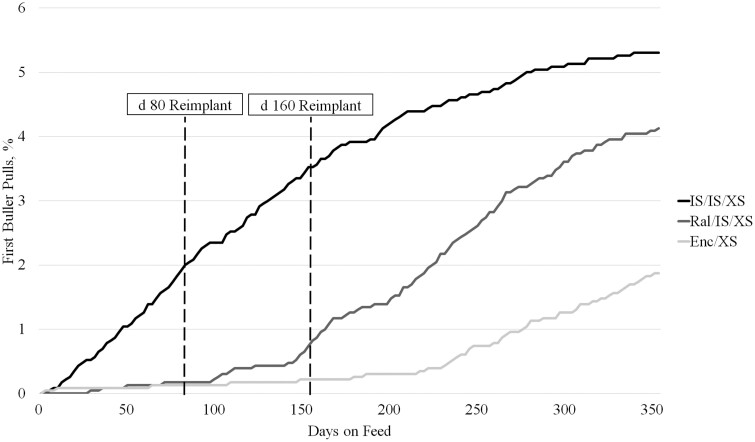
First buller pulls over days on feed of beef × dairy steer administered different implant programs and terminally sorted. Only first buller pulls are represented in the above chart. Implant treatments consisted of Revalor-IS (80 mg TBA + 16 mg E_2_; IS; Merck Animal Health, Madison, NJ) administered on d 0 and d 80, and Revalor-XS (200 mg TBA + 20 mg E_2_; XS; Merck Animal Health) on d 160 (**IS/IS/XS**); Ralgro (36 mg zeranol; Merck Animal Health) administered at d 0, IS at d 80, and XS at d 160 (**Ral/IS/XS**); or Encore (43.9 mg E_2_; Elanco Animal Health, Greenfield, IN) administered at d 0 and XS at d 160 (**Enc/XS**). Steers were terminally sorted within block and treatment into light, medium, or heavy groups between d 244 and d 300.

No difference in buller pulls was observed in the first 80 days following initial implant (*P* = 0.25), but from d 80 to 160 IS/IS/XS had the most pulls (*P* < 0.01). Buller pulls were similar (*P* > 0.05) between IS/IS/XS and Ral/IS/XS, both of which were greater (*P* < 0.01) than Enc/XS between d 160 to the completion of the study. First buller pulls were greatest (*P* < 0.01) for IS/IS/XS and Ral/IS/XS (5.27% and 4.16% vs. 1.90%; Figure 2) but were not different (*P* = 0.25) in the first 80 days. From d 80 to d 160, IS/IS/XS had the greatest (*P* < 0.01) percentage of first pull bullers, while Ral/IS/XS had the most (*P* = 0.03) first pulls from d 160 to the conclusion of the study. Percentage of bullers is similar to those reported by Kayser et al. (2019) and lower than those observed by Quinn et al. (2020). Kayser et al. (2019) reported the greatest percentage of bullers in the medium dose and timing of TBA administration treatment, contrary to the current study where percentage of bullers increased linearly with TBA potency and timing of administration. Similar to the outcome observed in the current study, Quinn et al. (2020) reported few to no buller treatments from d 0 to first reimplant, and the greatest increase of buller treatments after 160 days on feed.

### Live Performance (Mortalities and Removals Included)

Live performance outcomes including deads and removals are presented in Table 3. By design, no difference (*P* = 0.50) in initial BW due to implant treatment was observed. At administration of final implant on d 160, BW was different (*P* = 0.03) across implant treatments; Ral/IS/XS had increased (*P* < 0.05) BW compared to Enc/XS steers, and IS/IS/XS was intermediate. Final BW was similar (*P* = 0.78) across implant treatments. Scheffler et al. (2003) indicated an increased concentration of growth-promoting implants was associated with greater final body weight. Therefore, researchers expected steers administered IS/IS/XS to have heavier final body weights than Ral/IS/XS and Enc/XS. Although not statistically different, the least mortality rates were observed in the Ral/IS/XS treatment and allowed for less penalization of final BW from dead and removed animals. This may have contributed to improved performance of Ral/IS/XS on a deads and removals included basis over the greatest hormone concentration treatment of IS/IS/XS.

**Table 3.** Deads and removals included (DRI), live performance data of beef × dairy steers administered differing implant programs

Item	Treatment^1^				*P*-value
	IS/IS/XS	Ral/IS/XS	Enc/XS	SEM	Implant
Allotment pens, *n*	30	30			
Initial BW, kg	156	157	157	1.7	0.50
D160 BW, kg	380^ab^	384^a^	375^b^	5.5	0.03
Final BW, kg	585	586	581	8.6	0.78
DMI, kg/hd/d					
D0–D160	6.92	6.89	6.86	0.784	0.33
D160–End	9.81^a^	9.64^ab^	9.46^b^	0.085	0.01
D0–End	8.54^a^	8.47^ab^	8.35^b^	0.063	<0.01
ADG, kg/d					
D0–D160	1.46^a^	1.48^a^	1.42^b^	0.028	<0.01
D160–End	1.46	1.38	1.42	0.030	0.17
D0–End	1.42	1.41	1.39	0.016	0.12
G:F					
D0–D160	0.211^ab^	0.214^a^	0.208^b^	0.003	<0.01
D160–End	0.149^jk^	0.143^k^	0.150^j^	0.002	0.09
D0–End	0.167	0.166	0.167	0.002	0.92

^1^IS/IS/XS = Revalor-IS (80 mg TBA and 16 mg estradiol; Merck Animal Health) on d 0 and d 80, and Revalor-XS (200 mg TBA and 40 mg estradiol; Merck Animal Health) on d160; Ral/IS/XS = Ralgro (36 mg zeranol; Merck Animal Health) on d 0, Revalor-IS on d 80, and Revalor-XS on d 160; Enc/XS = Encore (43.9 mg estradiol; Elanco Animal Health) on d 0 and Revalor-XS on d 160.

^a,b,c^Means in a row with varying superscripts differ (*P* < 0.05).

^j,k^Means in a row with varying superscripts differ (*P* < 0.10).

**Figure 3.**
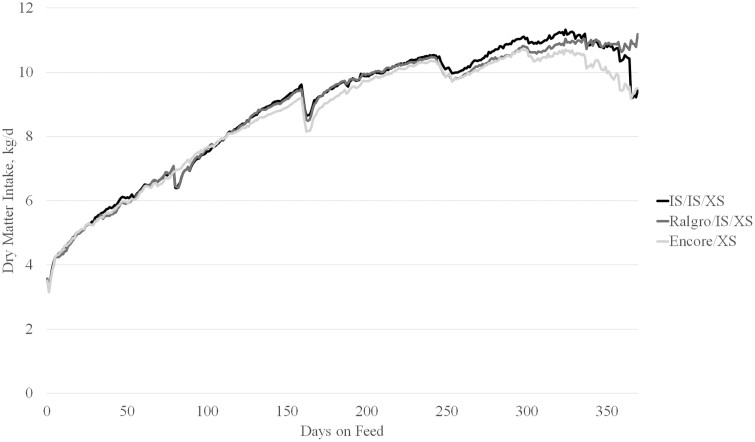
Daily dry matter intake in kg/d over days on feed of beef × dairy steer administered different implant programs and terminally sorted. Partial feedings to satiate cattle prior to afternoon shipment to beef packer are included in the above chart, final shipment dates were between d 281 and d 370. Implant treatments consisted of Revalor-IS (80 mg TBA + 16 mg E_2_;d IS; Merck Animal Health, Madison, NJ) administered on d 0 and d 80, and Revalor-XS (200 mg TBA + 20 mg E_2_; XS; Merck Animal Health) on d 160 (**IS/IS/XS**); Ralgro (36 mg zeranol; Merck Animal Health) administered at d 0, IS at d 80, and XS at d 160 (**Ral/IS/XS**); or Encore (43.9 mg E_2_; Elanco Animal Health, Greenfield, IN) administered at d 0 and XS at d 160 (**Enc/XS**). Steers were terminally sorted within block and treatment into light, medium, or heavy groups between d 244 and d 300.

Dry matter intake (**DMI**) was recorded on a pen basis and averaged for number of animals in the pen and, therefore, is only presented on a deads and removals included analysis (Figure 3). In the first 160 days of the trial, there was no difference (*P* = 0.33) in DMI as a result of implant treatment. From d 160 to completion of the trial, IS/IS/XS steers had greater DMI (*P* < 0.05) than Enc/XS steers, while Ral/IS/XS was intermediate (*P* = 0.01). Overall, steers which received IS/IS/XS consumed more (*P* < 0.01) feed than those administered Enc/XS, and Ral/IS/XS was intermediate (8.54 vs. 8.35 and 8.47 kg/d). In agreement with the literature, an increased DMI was associated with increased potency of combination TBA and E_2_ implant administration (Scheffler et al., 2003; Smith et al., 2018).

In the first 160 days on trial, steers which received a Revalor-IS implant prior to d 160 (IS/IS/XS and Ral/IS/XS) had 6.5% increased (*P* < 0.05) average daily gain (**ADG**) compared to those which received Enc/XS (1.46 and 1.48 vs. 1.42 kg/d; *P* = 0.01). No differences in ADG were observed during the second half of the trial (*P* = 0.17), or overall (*P* = 0.12). Similarly, Scheffler et al. (2003) reported steers administered an implant early in the feeding period increased ADG halfway through the study compared to those not administered a TBA and estradiol implant. In contrast to the current study, an early and greater total potency of combination implant administration increased overall ADG (Scheffler et al., 2003; Torenterra et al., 2016); however, the present study was conducted over a longer feeding period and could have resulted in diminished responses to implant administration due to extended days on feed. Although not significant, numerical differences suggest increased total potency of growth promoting implants can increase overall ADG.

Gain to feed efficiency was improved (*P* < 0.01) 3.4% in the first 160 days of the trial for Ral/IS/XS compared to Enc/XS steers, while IS/IS/XS steers were intermediate (0.214 vs. 0.208 and 0.211 units). A tendency (*P* = 0.09) for improved gain to feed efficiency was observed for IS/IS/XS and Enc/XS over Ral/IS/XS from d 160 to completion of the study (0.149 and 0.150 vs. 0.214 units). However, no differences (*P* = 0.92) in gain to feed efficiency were detected overall. These results agree with Scheffler et al. (2003), who reported overall gain to feed efficiency was similar in Holstein steers administered the same final implant on the same day, regardless of initial implant timing or total concentration of hormones. Although limited research has been conducted to determine the cause of feed efficiency similarities across breeds, Perry et al. (1991) noted Holstein cattle were less impacted by implant administration than Angus cattle. Researchers speculate light muscling, little external fat, and high energy requirements may alter hormone receptor density or sensitivity and cause Holstein-influenced cattle to be less responsive to implants than traditional beef animals.

### Live Performance (Mortalities and Removals Excluded)

Live performance outcomes are presented on a deads and removals excluded basis in Table 4. Body weight at d 160 was affected (*P* < 0.01) by implant treatment. Such that steers administered a Revalor-IS at d 0 or d 80 (Ral/IS/XS and IS/IS/XS) increased BW 1.5% compared to steers administered Enc/XS that did not receive a combination TBA and estrogen implant until d 160 (410 and 412 vs. 405 kg). Research has consistently demonstrated that both beef- and dairy-type steers increase body weight following implant administration (Scheffler et al., 2003; Parr et al., 2011; Quinn et al., 2020), and differences in body weight between implanted and non-implanted steers can be observed as early as d 70 post-implantation (Smith et al., 2018). Final BW was not different (*P* = 0.12) as a result of implant treatment. Although some researchers report increased potency and number of implants administered to cattle resulted in greater final BW (Scheffler et al., 2003; Parr et al., 2011; Smith et al., 2018), findings of the present study more closely agree with researchers who reported no differences in final BW of dairy-type steers administered various initial and terminal implant programs (Kayser et al., 2019; Quinn et al., 2020).

**Table 4.** Deads and removals out (DRO), live performance data of beef × dairy steers administered differing implant programs

Item	Treatment^1^				*P*-value
	IS/IS/XS	Ral/IS/XS	Enc/XS	SEM	Implant
Allotment pens, *n*	30	30	30		
Initial BW, kg	156	157	157	1.7	0.50
D160 BW, kg	412^a^	410^a^	405^b^	4.3	<0.01
Final BW, kg	659	652	651	8.2	0.12
ADG, kg/d					
D0–D160	1.60^a^	1.58^a^	1.55^b^	0.023	<0.01
D160–End	1.46	1.44	1.46	0.021	0.50
D0–End	1.53^j^	1.51^jk^	1.50^k^	0.014	0.08

^1^IS/IS/XS = Revalor-IS (80 mg TBA and 16 mg estradiol; Merck Animal Health) on d 0 and d8 0, and Revalor-XS (200 mg TBA and 40 mg estradiol; Merck Animal Health) on d160; Ral/IS/XS = Ralgro (36 mg zeranol; Merck Animal Health) on d 0, Revalor-IS on d 80, and Revalor-XS on d 160; Enc/XS = Encore (43.9 mg estradiol; Elanco Animal Health) on d 0 and Revalor-XS on d 160.

^a,b,c^Means in a row with varying superscripts differ (*P* < 0.05).

^j,k^Means in a row with varying superscripts differ (*P* < 0.10).

Average daily gain was different (*P* < 0.01) in the first 160 days as a result of implant treatment. Steers administered TBA-containing implants (IS/IS/XS or Ral/IS/XS) before d 160 improved (*P* < 0.05) ADG 2.5% over steers administered Enc/XS (1.60 and 1.58 vs. 1.55 kg/d). Average daily gain of all treatments after Revalor-XS administration on d 160 was similar (*P* = 0.50) regardless of initial implant treatment. Overall, ADG tended to be different (*P* = 0.08) greatest for IS/IS/XS steers. Increased implant potency is commonly associated with increased ADG compared to non-implanted cattle (Scheffler et al., 2003; Torrentera et al., 2016) with the differences observed as soon as 70 days after implant administration (Smith et al., 2018). In the present study, overall ADG tended to be increased by increased implant potency.

### Carcass Characteristics

Carcass characteristics are presented in Table 5. Dressing percentage was similar (*P* = 0.28) across implant treatments. These findings agree with Quinn et al. (2020) and Kayser et al. (2019), who both reported no differences in dressing percentage of steers administered different timing or total potency of TBA.

**Table 5.** Carcass characteristics of beef × dairy steers administered differing implant programs

Item	Treatment^1^				*P*-value
	IS/IS/XS	Ral/IS/XS	Enc/XS	SEM	Implant
Terminal sort pens, *n*	90	90	90		
Number of carcasses	2,001	2,023	2,012		
Carcass-adj final body weight, kg	657	653	651	9.25	0.76
Dressing percentage, %	63.1	63.3	63.4	0.13	0.28
Hot carcass weight, kg	416	413	412	5.85	0.76
Camera data					
Longissimus muscle area, cm sq	91.7	91.0	90.4	0.89	0.58
Marbling score^2^	444^k^	446^jk^	460^j^	8.5	0.09
12th rib fat thickness, cm	1.22	1.20	1.19	0.045	0.60
USDA Quality Grade					
Prime, %	0.9^b^	0.9^b^	2.0^a^	0.62	<0.01
Choice, %	68.8	69.5	70.9	3.07	0.34
Select, %	28.7^a^	26.9^ab^	23.9^b^	3.30	<0.01
Certified Angus Beef, %	18.2^b^	20.6^ab^	22.5^a^	2.55	<0.01
USDA Yield Grade					
YG 1, %	8.9^k^	10.9^j^	10.3^jk^	1.82	0.09
YG 2, %	47.3	47.6	47.9	2.29	0.93
YG 3, %	36.0	33.1	33.6	2.91	0.12
YG 4 and 5, %	5.2	5.5	5.5	1.28	0.87
EBF^2^, %	29.3	29.2	29.1	0.31	0.67

^1^IS/IS/XS = Revalor-IS (80 mg TBA and 16 mg estradiol; Merck Animal Health) on d 0 and d 80, and Revalor-XS (200 mg TBA and 40 mg estradiol; Merck Animal Health) on d 160; Ral/IS/XS = Ralgro (36 mg zeranol; Merck Animal Health) on d 0, Revalor-IS on d 80, and Revalor-XS on d 160; Enc/XS = Encore (43.9 mg estradiol; Elanco Animal Health) on d 0 and Revalor-XS on d 160.

^2^Small = 400–499, Modest = 500–599, Moderate = 600–699.

^3^Calculated using Guiroy et al. (2002)

^a,b,c^Means in a row with varying superscripts differ (*P *< 0.05).

^j,k^Means in a row with varying superscripts differ (*P* < 0.10).

No difference (*P* = 0.76) in carcass-adjusted final BW was observed due to implant treatment. Smith et al. (2018) reported differences in carcass-adjusted final BW across implant treatments with different TBA release patterns in beef steers, as well as differences in final BW and dressing percentage. The present study did not observe differences in dressing percentage or final BW, which was likely attributed to similarities in dressing percentage among implant treatments.

No difference in HCW was observed among implant treatments (*P *= 0.76). Similar studies which evaluated total potency and timing of TBA administration reported increased HCW with earlier and more TBA administered (Scheffler et al., 2003; Kayser et al., 2019; Quinn et al., 2020). Differences from the published literature could be attributed in part to a variation in HCW due to different days on feed within sort groups (heavy, medium, and light sort group average days on feed at shipment was 309 ± 19, 329 ± 19, and 348 ± 19 days) across blocks.

Steers Longissimus muscle area (**LMA**) did not differ by treatment (*P* = 0.58). Increased LMA as a result of increased potency of TBA has been observed in multiple trials with implanted steers compared to non-implanted controls (Scheffler et al., 2003; Parr et al., 2011; Torrentera et al., 2016). However, present results are more aligned with Smith et al. (2018), who reported timing of TBA administration, given similar potencies in beef steers, did not affect LMA outcomes.

Marbling score tended to be different (*P* = 0.09) across implant treatments. Steers administered Enc/XS had improved marbling score 3.5% compared to steers given IS/IS/XS, and Ral/XS were intermediate (460 vs. 444 and 446 points). Scheffler et al. (2003) and Smith et al. (2018) reported the greatest marbling score for cattle not administered any implants. In the present study, steers administered Enc/XS received the least TBA and had the greatest marbling score.

Fat thickness measured at the 12th rib was similar (*P* = 0.60) across implant treatments. Torrentera et al. (2016) and Scheffler et al (2003) also reported no differences in 12th rib fat thickness for steers administered differing potencies or administration time of implants.

Empty body fat (**EBF**) percentage as calculated by Guiroy et al., (2002) was similar (*P* = 0.67) across all treatments. These results are similar to findings from Smith et al. (2018) who reported no differences in EBF percentage in cattle administered similar drug combination and dose of implants at various time points.

Carcasses graded USDA Prime were greatest (*P* < 0.01) for Enc/XS treatment and similar between IS/IS/XS and Ral/IS/XS treatments (2.0% vs. 0.9% and 0.9%). Percentage of carcasses which graded USDA Choice were similar (*P* = 0.34) across implant treatments. Percentage of USDA Select carcasses was least *(P* < 0.01) in Enc/XS treatments compared to Ral/IS/XS and IS/IS/XS treatments (23.9% vs. 26.9% and 28.7%). Carcasses which qualified for Certified Angus Beef (**CAB**) specification must meet several requirements, one of which includes presenting a marbling score of Modest or Moderate (commonly referred to as upper two-thirds Choice). Carcasses which graded CAB were greatest *(P* < 0.01) from Enc/XS treatment and fewest in IS/IS/XS treated cattle (22.5% vs. 18.2%). These results indicate although there was no difference in percentage of USDA Choice carcasses overall, Enc/XS steers graded a greater percentage of upper two-third Choice compared to other implant treatments. Results agree with similar studies that reported increased percentage USDA Prime or Choice and decreased percentage of USDA Select in steers administered lower or delayed potencies of TBA (Parr et al., 2011; Kayser et al., 2019; Quinn et al., 2020). Other researchers have reported detrimental effects of TBA on marbling development (Chung et al., 2012; Smith et al., 2017).

Yield grade outcomes were similar (*P* ≥ 0.12) across implant treatments. Only carcasses which graded YG 1 tended (*P* = 0.09) to be different across treatments, such that Ral/IS/XS and Enc/XS steers had the greatest proportion of YG 1 carcasses (10.9% and 10.3% vs. 8.9%). Numerically, Ral/IS/XS and Enc/XS carcasses were less likely to grade YG 3, because they were more likely to fall into YG 1 classifications. Outcomes for YG in response to implant treatment are inconsistent in published literature, where some researchers report a greater percentage of YG 1 or 2 carcasses in steers administered earlier or greater amounts of TBA (Parr et al., 2011; Smith et al., 2018). Others reported no implant differences in YG (Parr et al., 2011), or observed differences were not easily interpreted by treatments (Kayser et al., 2019).

### Liver Scores

All liver outcomes are presented in Table 6. Percentage of edible livers tended (*P* = 0.06) to be different for implant treatments, where Enc/XS had a greater percentage of edible livers than Ral/IS/XS and IS/IS/XS was intermediate (34.9% vs. 31.3% and 33.1%). This observation is in contrast to Parr et al., 2011 and Kayser et al., 2019, both of whom reported no implant differences in proportion of edible livers. An interaction between liver abscess occurrence and implant regimen is not biologically likely, given lack of correlation to steroid potency and similarities in dry matter intake. Therefore, differences in edible livers observed in the present study could represent a type I error.

**Table 6.** Liver scores of beef × dairy steers administered differing implant programs

Item	Treatment 1				*P-*value
	IS/IS/XS	Ral/IS/XS	Enc/XS	SEM	Implant
Livers, *n*	1851	2032	1877		
Edible, %	33.1^ab^	31.3^b^	34.9^a^	1.95	0.06
Abscessed, %	61.4^ab^	64.4^a^	60.5^b^	2.11	0.03
Severe Abscesses, %	26.8	28.3	27.6	1.62	0.56
A+	3.4	4.3	3.3	0.84	0.16
A + Adhered	14.9	14.9	15.2	1.47	0.97
A + Open^2^	8.6	9.6	9.1	1.04	0.65
Other, %	5.3	4.3	4.6	0.58	0.29

^1^IS/IS/XS = Revalor-IS (80 mg TBA and 16 mg estradiol; Merck Animal Health) on d 0 and d 80, and Revalor-XS (200 mg TBA and 40 mg estradiol; Merck Animal Health) on d 160; Ral/IS/XS = Ralgro (36 mg zeranol; Merck Animal Health) on d 0, Revalor-IS on d 80, and Revalor-XS on d 160; Enc/XS = Encore (43.9 mg estradiol; Elanco Animal Health) on d 0 and Revalor-XS on d 160.

^2^Includes both A + Open and A + Adhered/Open livers.

^a,b,c^Means in a row with varying superscripts differ (*P* < 0.05).

Occurrence of liver abscesses can lead to measurable reduction of live and carcass performance, especially in severely abscessed livers (Brink et al., 1990). Liver abscess occurrence, regardless of severity, was greatest (*P* = 0.03) in Ral/IS/XS and IS/IS/XS (64.4% and 61.4% vs. 60.5%). Amachawadi and Nagaraja (2016) stated Holstein steers were at a greater risk for liver abscesses and an increased proportion of severe liver abscess, and speculated this could be contributed to increased days on feed and energy intake. Moreover, Reinhardt and Hubbert (2015) reported Holstein steers fed in the high plains experienced a 55% occurrence of liver abscesses, and 48% severely abscessed livers. Grimes (2022) reported a nation-wide average of 50% abscessed livers in beef × dairy cattle and the greatest occurrences of liver abscesses in the Midwestern U.S. and Pacific Northwest. These data imply when dairy-type cattle are fed commercially in the high plains, there is increased variation in the rate liver abscesses, and cattle, in the present study, were among the upper end of the expect range. More research is needed to understand the cause of high liver abscess occurrence and is critical to the success of the beef × dairy cattle population in the cattle feeding industry.

Severe liver abscesses are presented as a percentage of all graded livers, and no differences in severe liver abscesses were observed as a result of implant treatment (*P* = 0.56). Furthermore, no differences in the occurrence of A+, A + Adhered, or A + Open liver abscesses were observed (*P* ≥ 0.16). Other liver outcomes which were classified as non-edible and non-abscessed, consisted of cirrhosis, flukes, and contamination, are presented as a percentage of all scored livers. No differences (*P* = 0.29) in other liver outcomes were observed for implant treatment.

## CONCLUSION

Morbidity, removals, and mortality outcomes were similar in regard to implant treatment. Total buller pulls were increased with increasing potency and earlier administration of TBA-containing implants. Live growth performance measured across the entire feeding period was similar across implant treatments. Hot carcass weight, adjusted final BW, LMA, and 12th rib fat thickness were not affected by implant treatment. Quality Grade improved in steers receiving lower potency and later administration of TBA. These results indicate delaying administration or decreasing total potency of TBA and estrogen combination implants may improve Quality Grade, with few differences in performance as a result of initial implant.
